# A current reference-enhanced strategy endows the GFC in DC microgrids better dynamic response

**DOI:** 10.1038/s41598-024-58928-5

**Published:** 2024-04-13

**Authors:** Wenqiang Xie, Xian Zheng, Mingming Shi, Jiaoxin Jia

**Affiliations:** 1 State Grid Jiangsu Electric Power Co. LTD. Research Institute, Nanjing, 211100 China; 2 North China Electric Power University, Baoding, 071003 China

**Keywords:** DC microgrids, Non-integrator control, Current reference enhancement, Dynamic performance, Load disturbances, Electrical and electronic engineering, Energy grids and networks

## Abstract

The constant voltage strategy (CVS) is more suitable for the small-capacity dc microgrid applications to form the dc bus voltage because it can eliminate the steady voltage deviation. However, its dynamic performance is slowed down by the integrator in the voltage loop and negatively influenced by the load steps. Some advanced methods have been conducted in the literature. However, their principle is to increase the system bandwidth, which is limited because the bandwidth is generally designed about one-tenth of the switching frequency and cannot be increased infinitely. This work pays efforts to increase the gain within the system bandwidth to accelerate the transient response and simultaneously eliminate the influence of the integrator. Therefore, the non-integrator disturbance observer-based (NIDOB) strategy is proposed in this work, and it can feed the load current into the current reference to replace the output of the integrator. Compared to the traditional and non-integrator directly-feedforward (NIDF) strategy, it has a better dynamic performance. Compared to the bandwidth-increased strategy, it does not introduce more noise. The theoretical analysis and experimental results prove its advantages.

## Introduction

The popularity of renewable energy and the increasing global demand for power consumption leads to the emergence of dc microgrids^1–3^. The storage converter plays the role of the grid-forming converter (GFC) in some small-capacity applications such as buildings and residences^4,5^, to form the bus voltage and balance the power variation caused by the renewable energy and load-steps^6,7^. In addition, only one GFC can be used in the applications operating in Island mode to form its bus voltage, and it is appropriate to adopt the constant voltage strategy (CVS)^8^. A multi-branch GFC can be utilized to increase the capacity of a single GFC to meet the requirements^9^. However, although the CVS can eliminate the steady voltage deviation, its transient response is limited because of the finite system bandwidth as well as the controller limitations.

The input voltage and load variations generally influence the dynamic performance of a GFC. In particular, the load-steps happen much more frequently than the input voltage variations. When a load-step happens, a voltage sag may occur immediately. Therefore, many strategies are explored to improve the dynamic performance of a GFC, such as the average current mode control^10^, current-programmed mode control^11,12^, and voltage mode control^13^. With these control schemes, the influence of the load-steps on the output voltage can be effectively reduced, but it may not be eliminated. The integrator also influences the dynamic performance in the voltage control loop because its output has to change continuously. There has been no work investigating such influence so far. Some studies adopt the feedforward strategy to accelerate the transient response^14^ adds an auxiliary loop to the original control plant, and^15^ design the input voltage feed-forward strategy to suppress the input voltage influence. However, the two approaches focus on the specific applications with wide input voltage variation, and the influence of load-steps cannot be eliminated^9^,^16^ design a feedforward loop to improve the dynamic response to the voltage sags, but its essence is to increase the bandwidth by adding an additional proportional loop. This approach requires a hysteresis comparator to avoid the negative influence on the steady performance. This method is relatively complicated and limited, since the larger bandwidth brings more noise^17^,^18^ propose the inductor current feed-forward strategy to suppress the disturbance of load-steps, but the inductor current cannot completely characterize the load current in the transient state.

The reasons for negatively influencing the dynamic performance of a GFC include the battery voltage variation, load-steps, and the integrator in the voltage loop. However, the battery voltage does not change frequently, and the changing process is very slow. Hence, this work focuses on eliminating the influence of load-steps and the integrator. In this study, it is found that the most effective approach to achieve these aims is to increase the gain within the designed system bandwidth. A new variable is discovered to replace the output of the integrator, rather than increasing the system bandwidth to allow more noise^19–21^. The domain within the system bandwidth is named the intermediate-frequency (IF) band, which refers to the frequency domain from zero to the cut-off frequency of the system. An easy method comes out that the load current is directly fed into the current reference (named integrator-included DF strategy) to replace the output of the integrator, which works effectively and increases the gain over the IF-band, but it needs an extra sensor. A soft method that can avoid using the extra sensor is to design an observer to acquire the load current and then to feed it as the current reference^22,23^. The method operates by lumping all the internal uncertainties and external disturbances as a whole and then estimates that through an observer. Then the observation value can be transmitted into the baseline for an advanced controller^24^. Based on these merits, the non-integrator disturbance observer-based (NIDOB) strategy is proposed to realize the CVS and improve the dynamic performance of a GFC.

This work contributes to proposing a novel CVS, which can quickly respond to load disturbances and eliminate the influence of the integrator. The NIDOB strategy is proposed and designed in detail to achieve these two aims The gain over the IF band is increased but the bandwidth is not enlarged, so that the dynamic performance can be improved without introducing more noise consequences. Further theoretical analysis shows that the feedforward observation can well play the role of the current reference, and the output of the integrator in the voltage loop can converge to the steady zero. Therefore, the integrator can be directly removed. The NIDOB strategy is compared to the traditional and the non-integrator DF (NIDF) strategy. The locus comparison shows that the NIDOB strategy has the fastest dynamic performance and largest stability margin. In addition, it is also compared to the bandwidth-increased strategy^19,20^, and the result indicates that the NIDOB strategy has a larger gain over the IF domain than the bandwidth-increased method. It can be concluded that it has a better dynamic performance under the same bandwidth unless the latter tolerates more noises.

This paper is structured as follows. Section 2 constructs the system model from the controller and physical system perspectives, then analyzes the reasons for the slow transient response of a GFC. Section 3 proposes two feasible methods and compares them with each other, and then finds out the NIDOB strategy can endow a GFC with better dynamic performance. Section 4 theoretically verifies the correctness of removing the integrator in the NIDOB strategy, and compares it to the traditional, NIDF, and bandwidth-increased strategies. Experimental results are presented to prove the advantages of the NIDOB strategy in Sect. 5. Finally, conclusions are drawn in Sect. 6.

## Problem formulation

**Figure 1:**
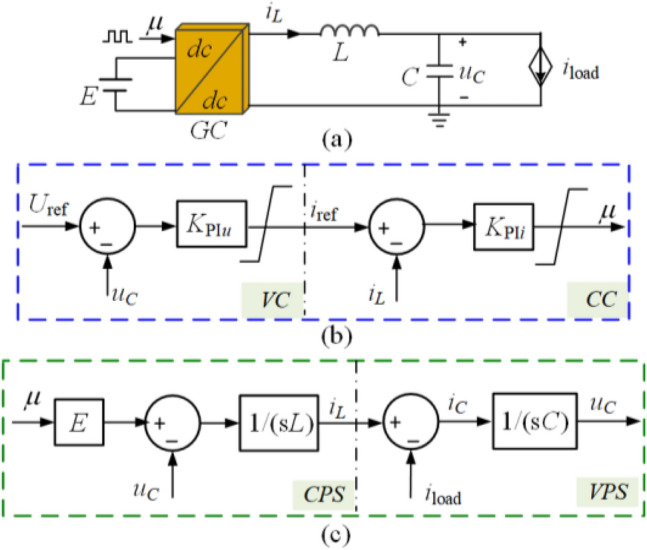
The traditional CVC system. (**a**) An employed circuit of GFC. (**b**) The diagram of controller. (**c**) The physical system.

The traditional CVS employed in a GFC can be depicted in Fig. 1, which consists of four units, the voltage controller (VC), current controller (CC), current physical system (CPS), and voltage physical system (VPS). VC and VPS constitute the voltage loop, and CC and CPS constitute the current loop. From Fig. 1, it can be seen that the two proportional-integral (PI) controllers are included, where the inner PI controller (*K*_PI*i*_) for CC and the outer PI controller (*K*_PI*u*_) for VC, respectively. The two PI controllers are separately expressed as

1 $$K_{{{\text{PI}}u}} = k_{{{\text{pu}}}} + {{k_{{{\text{iu}}}} } \mathord{\left/ {\vphantom {{k_{{{\text{iu}}}} } s}} \right. \kern-0pt} s}, \, K_{{{\text{PI}}i}} = k_{{{\text{pi}}}} + {{k_{{{\text{ii}}}} } \mathord{\left/ {\vphantom {{k_{{{\text{ii}}}} } s}} \right. \kern-0pt} s}.$$

where *k*_pu_ and *k*_iu_ are the proportional and integral parameters in VC, *k*_pi_ and *k*_ii_ are the proportional and integral parameters in CC, respectively.

In this traditional CVS, the output can be depicted as

2 $$u_{C} = G_{Uu} U_{{{\text{ref}}}} + G_{iu} i_{{{\text{load}}}} ,$$

where

3 $$\left\{ \begin{gathered} G_{Uu} = \frac{{EK_{{{\text{PI}}u}} K_{{{\text{PI}}i}} }}{{sC\left( {sL + K_{{{\text{PI}}i}} E} \right) + EK_{{{\text{PI}}u}} K_{{{\text{PI}}i}} + 1}} \hfill \\ G_{iu} = \frac{{ - \left( {sL + K_{{{\text{PI}}i}} E} \right)}}{{sC\left( {sL + K_{{{\text{PI}}i}} E} \right) + EK_{{{\text{PI}}u}} K_{{{\text{PI}}i}} + 1}} \hfill \\ \end{gathered} \right..$$

Therefore, the bus voltage (*u*_*C*_) will be inversely influenced by the load current (*i*_load_). When a voltage variation happens, the *K*_PI*u*_ will generate the current reference (*i*_ref_) for the inner current loop to achieve voltage deviation-free. The *K*_PI*i*_ is utilized to help the output current (*i*_*L*_) track *i*_ref_, so as to speed the current response.

If practical applications show poor dynamical performance, it is mainly due to an improperly tuned current loop. Therefore^16^, improves the dynamic performance by increasing the proportional gain of the current loop. However, it increases the inner bandwidth, and reduces the ability of anti-disturbance. In terms of parameter design, the bandwidth of the inner loop is generally designed one-tenth of the switching frequency and wider than that of the outer loop. Following the above rule,* K*_PI*i*_ can tightly track *i*_ref_ and does not introduce important noises. The dynamic performance is up to whether the *K*_PI*u*_ can immediately generate a desired *i*_ref_. However, the transient response of an integrator is relatively slower than that of a pure proportional compensator. Therefore, this work focuses on generating the desired *i*_ref_ and removing the integrator, instead of increasing the bandwidth of the current loop. The reason for the relatively slow transient response of the traditional CVS is detailly analyzed as follows.

### The output of integrator changes continuously

The reason for the integrator in *K*_PI*u*_ slowing the dynamic performance is first explained. Since the *K*_PI*u*_ can achieve zero-deviation regulation, it can be considered that *i*_*L*_ = *i*_ref_ and *u*_*C*_ = *U*_ref_ in the steady-state. Assuming the system reaches a steady-state at *t* = *t*_1_, *i*_ref_ can be calculated as

4 $$i_{{{\text{ref}}}} \left( {t_{1} } \right) = \int_{0}^{{t_{1} }} {k_{{{\text{iu}}}} \left( {U_{{{\text{ref}}}} - u_{C} \left( t \right)} \right)} dt,$$

because the output of the proportional loop is 0. If a load-step happens at this time, the recovery time is needed before the bus voltage goes back to *U*_ref_.

Assuming a new steady-state time is *t*_2_, then

5 $$i_{{{\text{ref}}}} \left( {t_{{2}} } \right) = i_{{{\text{ref}}}} \left( {t_{1} } \right) + \int_{{t_{1} }}^{{t_{2} }} {k_{{{\text{iu}}}} \left( {U_{{{\text{ref}}}} - u_{C} \left( t \right)} \right)} dt.$$

It means that the system cannot immediately change from the last steady state to a new one. In other words, since the output of the integrator must change continuously, the instantaneous voltage response will be slowed down.

### Current reference limitedly respond the transient components of load disturbance

**Figure 2:**
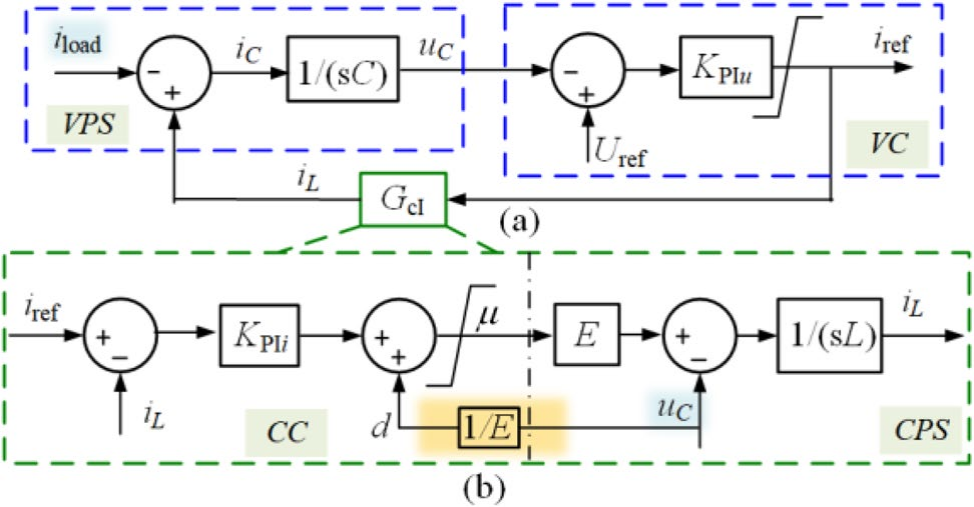
The decoupled control system. (**a**) The voltage loop. (**b**) The current loop.

From Fig. 1, the CPS is coupled with VPS by the *u*_*C*_, which makes it difficult to figure out the relationship between *i*_load_ and *i*_ref_. Therefore, a decoupling feedforward is added after *K*_PI*i*_ in CC to conveniently illustrate how the control plant slows the load disturbances transmission. The original current loop can be described as

6 $$i_{L} = \frac{{K_{{{\text{PI}}i}} E}}{{sL + K_{{{\text{PI}}i}} E}}i_{{{\text{ref}}}} + \frac{ - 1}{{sL + K_{{{\text{PI}}i}} E}}u_{C} ,$$

then, the feedforward can be designed as

7 $$d = {{u_{C} } \mathord{\left/ {\vphantom {{u_{C} } E}} \right. \kern-0pt} E}$$

to eliminate the influence of *u*_*C*_, where *d* denotes the decoupling feedforward. Therefore, the current loop can be described as

8 $$G_{{{\text{c}}I}} (s) = \frac{{K_{{{\text{PI}}i}} E}}{{sL + K_{{{\text{PI}}i}} E}}{ = }\frac{{k_{{{\text{pi}}}} s + k_{{{\text{ii}}}} }}{{{L \mathord{\left/ {\vphantom {L E}} \right. \kern-0pt} E}s^{2} + k_{{{\text{pi}}}} s + k_{{{\text{ii}}}} }},$$

and the decoupled control system is shown in Fig. 2.

From Fig. 2, the transfer properties from *i*_load_ to* i*_ref_ can be illustrated as

9 $$i_{{{\text{ref}}}} = \frac{{K_{{{\text{PI}}u}} }}{{sC + K_{{{\text{PI}}u}} G_{{{\text{c}}I}} \left( s \right)}}i_{{{\text{load}}}} + \frac{{sCK_{{{\text{PI}}u}} }}{{sC + K_{{{\text{PI}}u}} G_{{{\text{c}}I}} \left( s \right)}}U_{{{\text{ref}}}} .$$

It can be derived that

10 $$\mathop {\lim }\limits_{s \to 0} \frac{{sCk_{{{\text{PI}}u}} }}{{sC + k_{{{\text{PI}}u}} G_{{{\text{c}}I}} \left( s \right)}} = 0, \, \mathop {\lim }\limits_{s \to 0} \frac{{k_{{{\text{PI}}u}} }}{{sC + k_{{{\text{PI}}u}} G_{{{\text{c}}I}} \left( s \right)}} = 1.$$

It means the steady *i*_load_ can be fully responded by* i*_ref_. However, the load disturbance is a step-signal, which contains many transient components, expressed as

11 $$F\left[ {i_{{{\text{load}}}} } \right] = \left| {i_{{{\text{load}}}} } \right|\left( {\pi \delta \left( \omega \right) + \frac{1}{j\omega }} \right).$$

**Figure 3:**
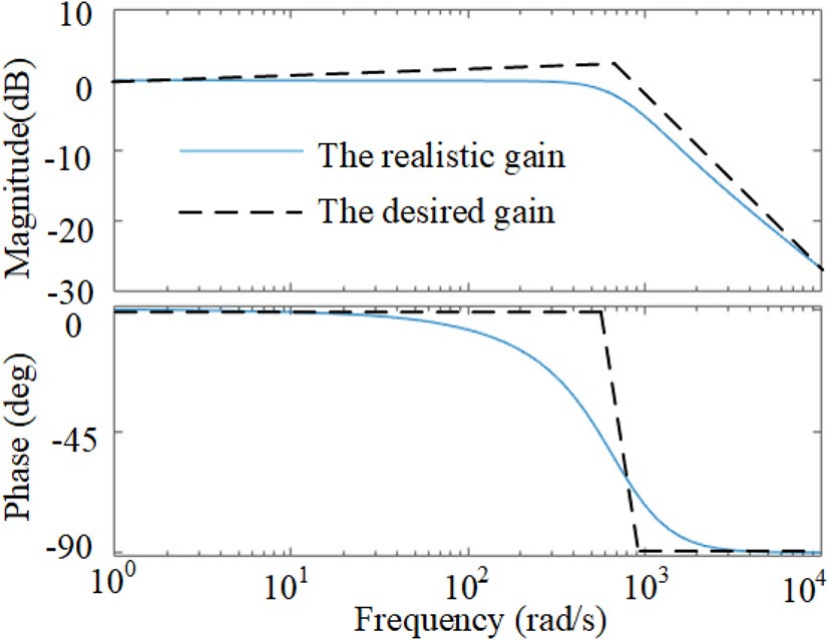
The frequency properties of the traditional CVS and the desired gain of an advanced strategy.

The current loop bandwidth is limited and only the IF components of *i*_load_ can be responded, as shown in Fig. 3, which results that the transient |*i*_ref_| is smaller than the desired value and the bus voltage recovers slowly. Therefore, the most effective approach is to increase the gain over the IF band to enlarge the *i*_ref_ as the black curve shows in Fig. 3, without the extra requirement for phase improvement. In this sense, two strategies are proposed and compared in Sect. 3.

## Proposed Strategies

In Fig. 2, it can be seen that *i*_ref_ is the output of *K*_PI*u*_ and the input of the current loop. But the steady component of *i*_ref_ is only generated by the integrator of *K*_PI*u*_. Therefore, it is necessary to make its steady output zero to remove the integrator. An effective approach is to find a new variable to replace it. In addition, the gain over the IF band should be increased to accelerate the transient response without introducing more noise. Two strategies are proposed as follows.

### The NIDF strategy

Considering *i*_ref_ = *i*_*L*_ = *i*_load_ in the steady state, *i*_L_ and *i*_load_ can possibly function as substitution variables. However, since *i*_L_ changes passively with *i*_load_, and the change is driven by the controller in GFC, it cannot instantly reflect the transient components of *i*_load_. Therefore, the approach that directly feedforwards *i*_load_ into *i*_ref_ is adopted. Besides, since *i*_load_ is equal to the steady output of the integrator, the integrator can be directly removed to achieve the non-integrator scheme. In this sense, the PI controller in the voltage loop can be simply designed as a proportional controller, which can be written in, *K*_PI*u*_ = *k*_pu_.

**Figure 4:**
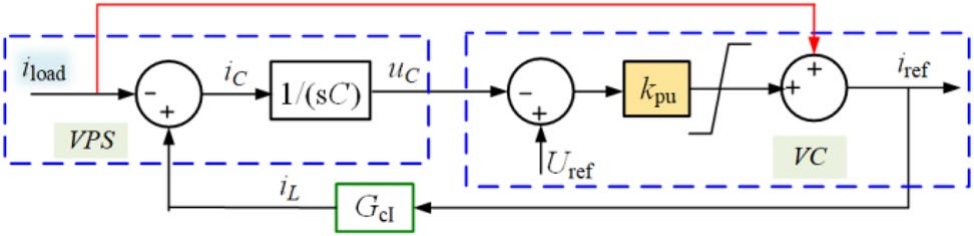
The NIDF strategy.

The NIDF strategy can be depicted in Fig. 4, and the current reference can be expressed as

12 $$i_{{{\text{ref}}}} = \frac{{sC + k_{{{\text{pu}}}} }}{{sC + k_{{{\text{pu}}}} G_{{{\text{c}}I}} \left( s \right)}}i_{{{\text{load}}}} + \frac{{sCk_{{{\text{pu}}}} }}{{sC + k_{{{\text{pu}}}} G_{{{\text{c}}I}} \left( s \right)}}U_{{{\text{ref}}}} .$$

It can be calculated that

13 $$\mathop {\lim }\limits_{s \to 0} \frac{{sC + k_{{{\text{pu}}}} }}{{sC + k_{{{\text{pu}}}} G_{{{\text{c}}I}} \left( s \right)}} = 1, \, \mathop {\lim }\limits_{s \to 0} \frac{{sCk_{{{\text{pu}}}} }}{{sC + k_{{{\text{pu}}}} G_{{{\text{c}}I}} \left( s \right)}} = 0.$$

It means that the NIDF strategy can effectively feed the load disturbance into *i*_ref_. Then, the output voltage can be expressed as

14 $$u_{C} = \frac{{k_{{{\text{pu}}}} G_{{{\text{c}}I}} \left( s \right)}}{{sC + k_{{{\text{pu}}}} G_{{{\text{c}}I}} \left( s \right)}}U_{{{\text{ref}}}} + \frac{{G_{{{\text{c}}I}} \left( s \right) - 1}}{{sC + k_{{{\text{pu}}}} G_{{{\text{c}}I}} \left( s \right)}}i_{{{\text{load}}}} .$$

It can be derived that

15 $$\mathop {\lim }\limits_{s \to 0} \frac{{k_{{{\text{pu}}}} G_{{{\text{c}}I}} \left( s \right)}}{{sC + k_{{{\text{pu}}}} G_{{{\text{c}}I}} \left( s \right)}} = 1, \, \mathop {\lim }\limits_{s \to 0} \frac{{G_{{{\text{c}}I}} \left( s \right) - 1}}{{sC + k_{{{\text{pu}}}} G_{{{\text{c}}I}} \left( s \right)}} = 0.$$

Therefore, the NIDF strategy can achieve the constant voltage scheme, although the integrator in VC is removed. However, it can be also calculated that

16 $$\mathop {\lim }\limits_{s \to \infty } \frac{{sC + k_{{{\text{pu}}}} }}{{sC + k_{{{\text{pu}}}} G_{{{\text{c}}I}} }} = 1,$$

which means that the bandwidth of the NIDF strategy is infinite and more noises can be introduced, which is undesired. Besides, another drawback of this strategy is that it needs an extra sensor to measure *i*_load_, which increases the volume and investment cost of a GFC.

### The NIDOB strategy

**Figure 5:**
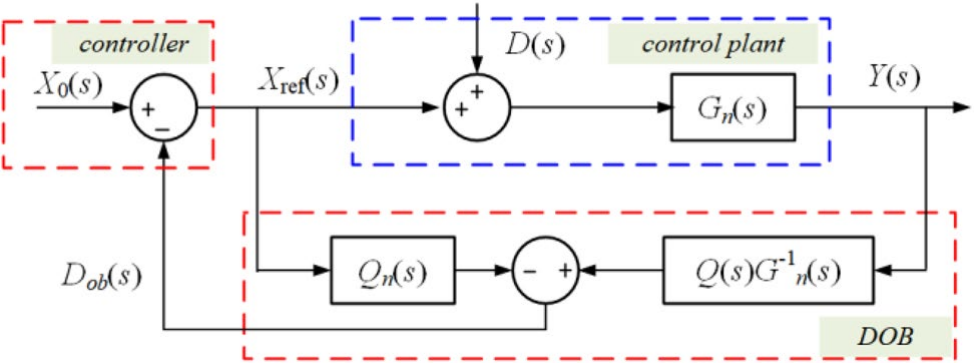
The typical structure of the observer application.

**Figure 6:**
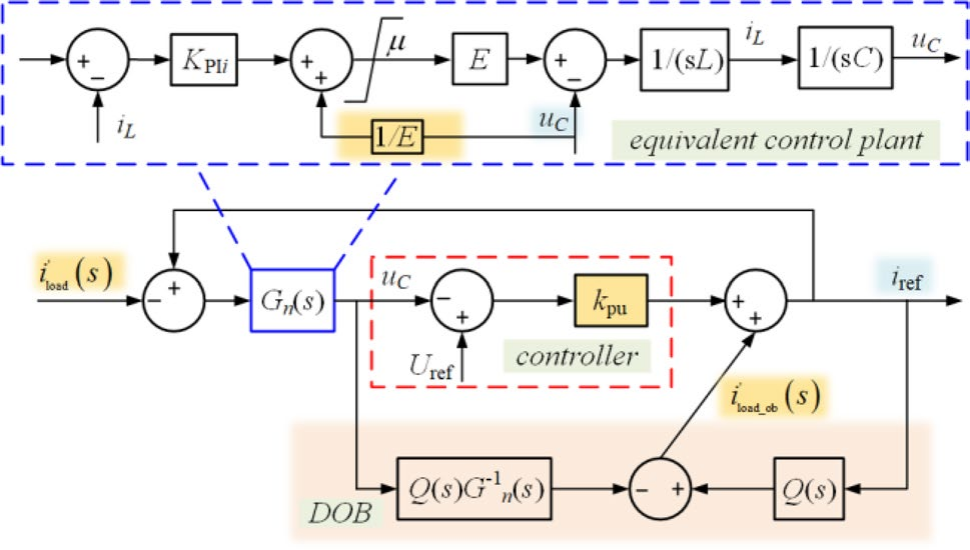
The NIDOB strategy.

The traditional observer-based method has been utilized in many applications^21–23^, which can observe the disturbance avoiding using an extra sensor. Since the observer can only be used in the specific structure as shown in Fig. 5, some structure changes of Fig. 2 are made as shown in Fig. 6. Besides, the non-integrator scheme is also achieved through rigid reasoning and design.

The principle of the traditional observer-based method can be explained as follows. In Fig. 5, *G*_*n*_(*s*) is the control plant and *X*_ref_(*s*) is its reference input, *X*_0_(*s*) comes from the controller, *Q*(*s*) is a low-pass filter,* D*(*s*) is the outer disturbance, and *D*_ob_(*s*) is the observation. The output of the original system without the observer can be expressed as

17 $$Y\left( s \right) = G_{n} \left( s \right)X_{0} \left( s \right) + G_{n} \left( s \right)D\left( s \right),$$

and it is influenced by the disturbance. When considering the feedforward of the observer, the output can be expressed as

18 $$Y\left( s \right) = G_{xy} \left( s \right)X_{0} \left( s \right) + G_{dy} \left( s \right)D\left( s \right),$$

where

19 $$G_{xy} \left( s \right) = \frac{{G_{n} \left( s \right)G_{n} \left( s \right)}}{{G_{n} \left( s \right) + Q\left( s \right)\left( {G_{n} \left( s \right) - G_{n} \left( s \right)} \right)}} = G_{n} \left( s \right)$$

and

20 $$G_{dy} \left( s \right) = \frac{{G_{n} \left( s \right)G_{n} \left( s \right)\left( {1 - Q\left( s \right)} \right)}}{{G_{n} \left( s \right) + Q\left( s \right)\left( {G_{n} \left( s \right) - G_{n} \left( s \right)} \right)}}.$$

Because *Q*(*s*) is a low-pass filter, it follows

21 $$\mathop {\lim }\limits_{s \to 0} G_{dy} \left( s \right) = 0.$$

Equation (19) implies that the transfer property from the controller to the output is not changed, and (21) implies that the influence of disturbance will be eliminated because it can be observed and fed into the reference to reject the physical change.

The observation can be represented as

22 $$\begin{aligned} D_{{{\text{ob}}}} \left( s \right) &= - Q(s)X_{{{\text{ref}}}} + \left( {X_{{{\text{ref}}}} + D\left( s \right)} \right)G_{n} \left( s \right)G_{n}^{ - 1} \left( s \right)Q(s) \\ &= Q(s)D\left( s \right). \\ \end{aligned}$$

Since *Q*(*s*) is a low-pass filter, the transient components over the high-frequency band will be filtered, and the components over the low and the IF band will be observed and fed. It can be derived that

23 $$\mathop {\lim }\limits_{s \to 0} D_{{{\text{ob}}}} \left( s \right) = \mathop {\lim }\limits_{s \to 0} \left( {Q(s)D\left( s \right)} \right) = \mathop {\lim }\limits_{s \to 0} D\left( s \right).$$

Therefore, the observer can be employed in CVS to feed *i*_load_ into *i*_ref_. Before this, it is necessary to figure out *G*_*n*_(*s*) and *X*_ref_(*s*) first, where *G*_*n*_(*s*) comes from the physical system, and *X*_ref_(*s*) comes from the controller.

Comparing Fig. 2 with Fig. 5, let

24 $$X_{{{\text{ref}}}} \left( s \right) = i_{{{\text{ref}}}} \left( s \right),$$

then, it is needed to move *G*_cI_(*s*) after *i*_load_ to constitute the equivalent control plant (*G*_*n*_(*s*)) together with VPS. Therefore, the equivalent diagram of the NIDOB strategy can be shown in Fig. 6, where the equivalent disturbance and its observation can be expressed as

25 $$\left\{ \begin{gathered} D\left( s \right) = - i_{{{\text{load}}}}^{\prime} \left( s \right) = - i_{{{\text{load}}}} \left( s \right)G_{{{\text{cI}}}}^{ - 1} \left( s \right) \hfill \\ D_{ob} \left( s \right) = - i_{{{\text{load\_ob}}}}^{\prime} \left( s \right) = - i_{{{\text{load\_ob}}}} \left( s \right)G_{{{\text{cI}}}}^{ - 1} \left( s \right) \hfill \\ \end{gathered} \right..$$

The equivalent control plant is

26 $$G_{n} \left( s \right) = \frac{{G_{{{\text{cI}}}} \left( s \right)}}{sC}{ = }\frac{{k_{{{\text{pi}}}} s + k_{{{\text{ii}}}} }}{{{{LC} \mathord{\left/ {\vphantom {{LC} E}} \right. \kern-0pt} E}s^{3} + Ck_{{{\text{pi}}}} s^{2} + Ck_{{{\text{ii}}}} s}}.$$

Substituting (8) into (23), it can be derived that

27 $$\left\{ \begin{gathered} \mathop {\lim }\limits_{s \to 0} i_{{{\text{load}}}}^{\prime} \left( s \right) = \mathop {\lim }\limits_{s \to 0} \left( {i_{{{\text{load}}}} \left( s \right)G_{{{\text{cI}}}}^{ - 1} \left( s \right)} \right) = i_{{{\text{load}}}} \hfill \\ \mathop {\lim }\limits_{s \to 0} i_{{{\text{load\_ob}}}}^{\prime} \left( s \right) = \mathop {\lim }\limits_{s \to 0} \left( {i_{{{\text{load\_ob}}}} \left( s \right)G_{{{\text{cI}}}}^{ - 1} \left( s \right)} \right) = i_{{{\text{load\_ob}}}} \hfill \\ \end{gathered} \right..$$

Therefore, the steady output of the integrator in the voltage loop can be replaced by the observation, and the integrator can be directly removed.

The output of the proposed NIDOB strategy can be expressed as

28 $$\begin{aligned} u_{C} \left( s \right) &= G_{Uu} \left( s \right)U_{{{\text{ref}}}} - G_{iu} \left( s \right)i_{{{\text{load}}}}{\prime} \left( s \right) \\&= G_{Uu} \left( s \right)U_{{{\text{ref}}}} - G_{iu} \left( s \right)G_{{{\text{cI}}}}^{ - 1} \left( s \right)i_{{{\text{load}}}} \left( s \right), \\ \end{aligned}$$

where

29 $$\begin{aligned} G_{Uu} \left( s \right) &= \frac{{k_{{{\text{pu}}}} \left( s \right)G_{n} \left( s \right)G_{n} \left( s \right)}}{{G_{n} \left( s \right) + Q\left( s \right)\left( {G_{n} \left( s \right) - G_{n} \left( s \right)} \right) + k_{{{\text{pu}}}} G_{n} \left( s \right)G_{n} \left( s \right)}} \\ &= \frac{{k_{{{\text{pu}}}} G_{n} \left( s \right)}}{{1 + k_{{{\text{pu}}}} G_{n} \left( s \right)}}, \\ \end{aligned}$$

30 $$\begin{aligned} G_{iu} \left( s \right)G_{{{\text{cI}}}}^{ - 1} \left( s \right) &= \frac{{G_{{{\text{cI}}}}^{ - 1} \left( s \right)G_{n} \left( s \right)G_{n} \left( s \right)\left( {1 - Q\left( s \right)} \right)}}{{G_{n} \left( s \right) + Q\left( s \right)\left( {G_{n} \left( s \right) - G_{n} \left( s \right)} \right) + k_{{{\text{pu}}}} G_{n} \left( s \right)G_{n} \left( s \right)}} \\ &= \frac{{G_{n} \left( s \right)\left( {1 - Q\left( s \right)} \right)}}{{G_{{{\text{cI}}}} \left( s \right) + k_{{{\text{pu}}}} G_{{{\text{cI}}}} \left( s \right)G_{n} \left( s \right)}}. \\ \end{aligned}$$

Because *Q*(*s*) is designed as a low-pass filter, (28) can be stated that

31 $$\mathop {\lim }\limits_{s \to 0} G_{du} \left( s \right)G_{{{\text{cI}}}}^{ - 1} \left( s \right) = 0,$$

and

32 $$\mathop {\lim }\limits_{s \to 0} G_{Uu} \left( s \right){ = }\frac{{k_{{{\text{pu}}}} k_{{{\text{pi}}}} s^{2} + k_{{{\text{ii}}}} k_{{{\text{pu}}}} }}{{\frac{LC}{E}s^{4} + Ck_{{{\text{pi}}}} s^{3} + \left( {Ck_{{{\text{ii}}}} + k_{{{\text{pu}}}} k_{{{\text{pi}}}} } \right)s^{2} + k_{{{\text{ii}}}} k_{{{\text{pu}}}} }}{ = }1.$$

Therefore, although the VC is designed as a proportional controller in the NIDOB strategy, the CVS can be achieved and the load disturbance will be effectively eliminated.

## Dynamic comparison

### Dynamic comparison of the proposed strategies

According to Fig. 6, the transfer property from *i*_load_ to *i*_ref_ can be expressed as

33 $$i_{{{\text{ref}}}} = \frac{{k_{{{\text{pu}}}} G_{n} \left( s \right) + Q\left( s \right)}}{{k_{{{\text{pu}}}} G_{n} \left( s \right) + 1}}i_{{_{{{\text{load}}}} }}^{\prime} + \frac{{k_{{{\text{pu}}}} }}{{k_{{{\text{pu}}}} G_{n} \left( s \right) + 1}}U_{{{\text{ref}}}} .$$

Substituting (23) into (30), it can be reformulated that

34 $$i_{{{\text{ref}}}} = \frac{{k_{{{\text{pu}}}} G_{n} \left( s \right) + Q\left( s \right)}}{{\left( {k_{{{\text{pu}}}} G_{n} \left( s \right) + 1} \right)G_{{{\text{cI}}}} \left( s \right)}}i_{{_{{{\text{load}}}} }} + \frac{{k_{{{\text{pu}}}} }}{{k_{{{\text{pu}}}} G_{n} \left( s \right) + 1}}U_{{{\text{ref}}}} .$$

According to the law of DOB^23^, since the relative degree of *G*_*n*_(s) is 2, the *Q*(*s*) can be designed as a two-order filter as

35 $$Q(s) = \frac{1}{{(\tau_{f} s{ + }1)^{2} }} = \frac{1}{{(\tau_{f} s)^{2} + 2\tau_{f} s{ + }1}},$$

and its cut-off frequency can be calculated by

36 $$\omega_{{\text{c}}} = (\sqrt 2 - 1)^{\frac{1}{2}} \frac{1}{{\tau_{f} }},$$

no more than the cut-off frequency (*ω*_s_) of the system, where *τ*_*f*_ is the time constant of *Q*(*s*). In this work, the parameters used in the following theoretical analysis and experiment are gathered in Table 1 in Sect. 5, where the switching frequency is 2 kHz, and the *ω*_s_ is designed about 1000 rad/s. The *τ*_*f*_ can be calculated by (36) no less than 0.7 ms, but the transient response will slow down as *τ*_*f*_ increases, hence *τ*_*f*_ is set as 2 ms in this work.

**Table 1:** Experimental parameters.

Parameter	*L* (mH)		*C*(m*F*)	*E*(V)	*k*_pu_	*k*_iu_	*k*_pi_	*k*_ii_
Value	2.2		1.8	200	1	2	0.01	0.1

**Figure 7:**
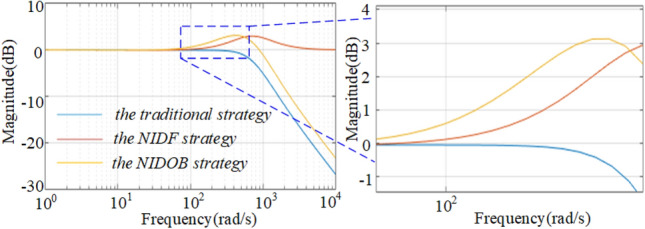
The gain comparison of the three methods over the IF band.

To illustrate the advantages of the NIDOB strategy, the gain of the current references of the three strategies are compared. Substituting (26) into (34) and then comparing it to (12), it can be found that, the components constituted by *U*_ref_ of (12) and (34) are the same but a bit larger than that of (9), because the integrator exists in the denominator of the components of (9). Therefore, it is needed to solely compare the transient components constituted by *i*_load_ in (34), (12), and (9). Hence, let

39 $$\left\{ \begin{gathered} c_{{{\text{tra}}}} = \frac{{K_{{{\text{PI}}u}} }}{{sC + K_{{{\text{PI}}u}} G_{{{\text{c}}I}} \left( s \right)}}i_{{{\text{load}}}} \hfill \\ c_{{{\text{DF}}}} = \frac{{k_{{{\text{pu}}}} + sC}}{{sC + k_{{{\text{pu}}}} G_{{{\text{c}}I}} \left( s \right)}}i_{{_{{{\text{load}}}} }} \hfill \\ c_{{{\text{DOB}}}} = \frac{{k_{{{\text{pu}}}} G_{cI} \left( s \right) + sCQ\left( s \right)}}{{G_{cI} \left( s \right)\left( {sC + k_{{{\text{pu}}}} G_{{{\text{c}}I}} \left( s \right)} \right)}}i_{{_{{{\text{load}}}} }} \hfill \\ \end{gathered} \right..$$

where* c*_tra_ denotes the component constituted by *i*_load_ in (9), *c*_DF_ denotes that in (12), and *c*_DOB_ denotes that in (34). The gains over the IF band of the three methods are compared in Fig. 7. It can be seen that the NIDOB strategy has the largest gain over the IF band whereas the traditional strategy has the smallest, which means the NIDOB strategy can respond faster to the load disturbance. In addition, the gain of the NIDOB strategy over the high-frequency band decreases rapidly to reject the noises, which solves the problem caused by the NIDF strategy.

### Stability comparison of the proposed strategies

**Figure 8:**
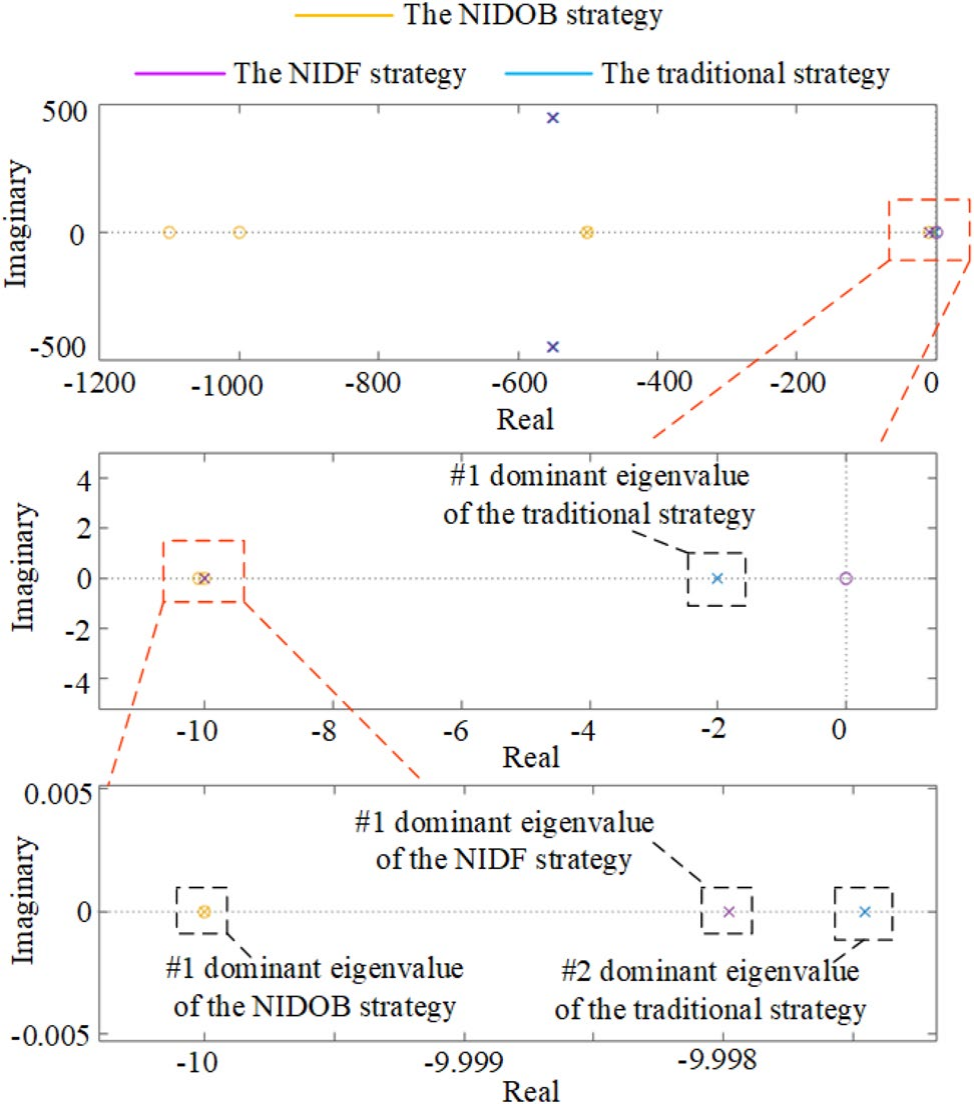
Stability comparison of the three strategies.

According to the parameters listed in Table 1 and the Eqs. (2), (14), and (28), the stability comparison can be rigidly verified by the locus distribution of the three strategies, as shown in Fig. 8. It can be seen that the traditional strategy has two dominant eigenvalues, but the NIDF strategy and the NIDOB strategy only have one dominant eigenvalue. Furthermore, the NIDOB strategy has the smallest dominant eigenvalue and it is almost offset by a zero, but the #1 dominant eigenvalue of the traditional strategy is close to zero. Therefore, the NIDOB strategy has the largest stability margin, but the traditional one has the smallest. In addition, the locus distribution also indicates that the NIDOB strategy has the fastest transient response, whereas the traditional one has the slowest, which is consistent with the dynamic comparison in Fig. 7.

### Comparison of the NIDOB strategy and the Bandwidth-Increased Strategy

**Figure 9:**
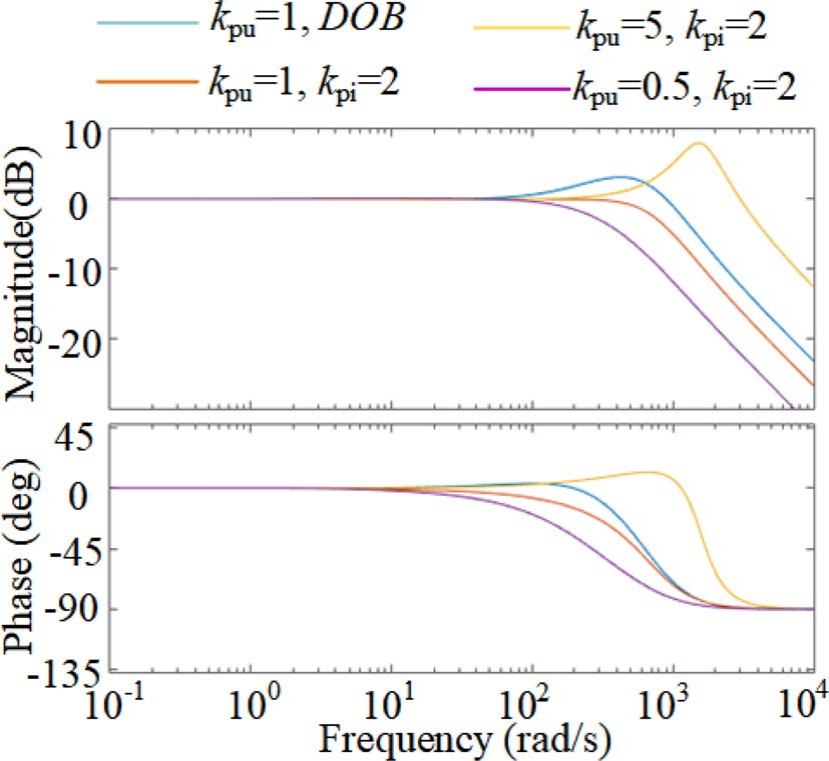
Dynamic comparison of the NIDOB strategy and the bandwidth-increased strategy.

Many works improve the dynamic performance by increasing the system bandwidth^19,20^, which may have a faster dynamic response when load-steps happen, but it cannot recover quickly due to the too many introduced noises. As shown in Fig. 9, with the increase of *k*_pu_, the bandwidth of the traditional strategy increases, much larger than that of the NIDOB strategy. However, the gain over the IF band of the NIDOB strategy is larger than that of the bandwidth-increased strategy. Hence, the NIDOB strategy has a better dynamic performance than the bandwidth-increased strategy under the condition of the same bandwidth.

### Sensitive analysis of the NIDOB strategy

Through analyzing the control structure in Fig. 6, it can be seen the stability of the proposed method is influenced by parameter *r*, *k*_p_, *k*_s_, *L*, *C*, and τ.

**Figure 10:**
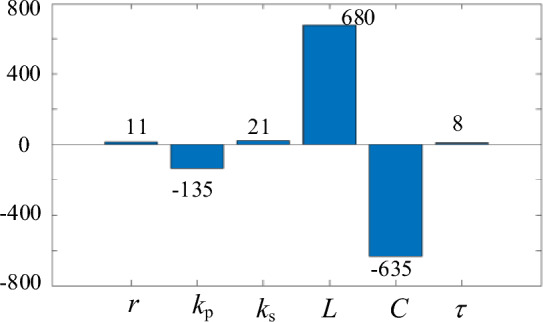
Sensitivity analysis of the real part of the dominant eigenvalue.

The sensitivity analysis is shown in Fig. 10. The positive number indicates that the parameter will cause the eigenvalues to shift to the right. On the contrary, the negative number indicates that the parameter will cause the eigenvalues to shift to the left. It can be seen that the increasement of *r*, *k*_s_, *L*, and τ will weaken the stability and the L influence the most. The inductor* L* will in the equipment are not easily changed, but the control parameter *r*, *k*_s_, and τ is adjustable. Therefore, *r*, *k*_s_, and τ should be set a relatively small value in parameter design.

## Experimental results

**Figure 11:**
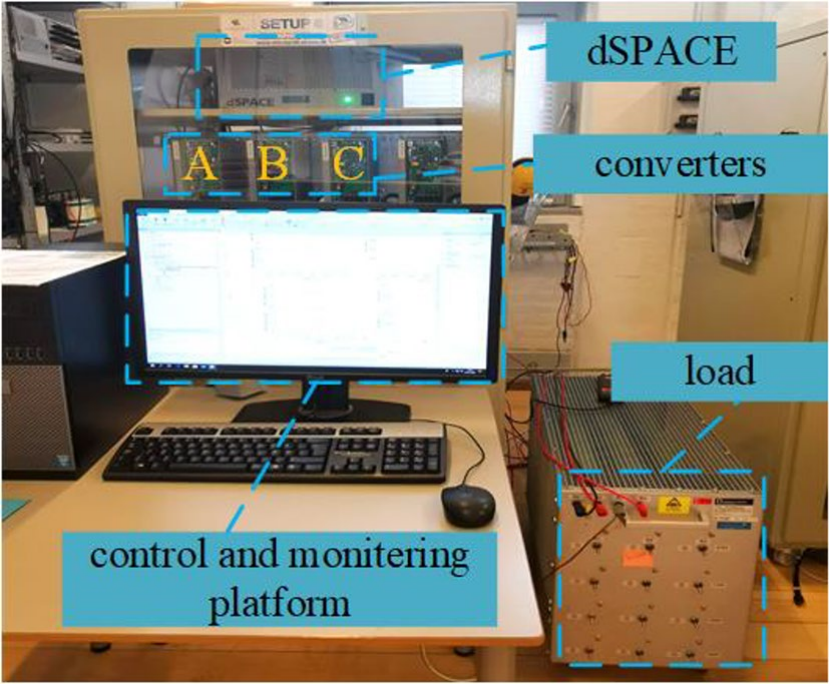
Experimental setup.

An experimental islanded dc microgrid setup, shown in Fig. 11, was used to verify the effectiveness of proposed strategy. The experimental system consists of a dc source, three DC/DC converters (GFCs), LC filters, programmed loads, and a dSPACE controller as well as its monitoring platform. The DC source voltage is 200 V, and the output voltage reference of DC/DC converter is set as 100 V. The sampling frequency of dSPACE controller is 10 kHz, and the switching frequency of DC/DC is set as 1 kHz. The other experimental parameters are shown in Table 1 after stability.

The three DC/DC converters separately adopt different strategies to make comparisons in experiment cases. In case 1, converter A adopts the NIDF strategy, converter B adopts the integrator-included DF strategy, and converter C adopts the traditional CV strategy. In case 2, converter A adopts the NIDF strategy, converter B adopts the NIDOB strategy, and converter C adopts the DOB-based strategy with integrator. In case 3, only two converters are used. The converter A adopts the bandwidth-increased strategy, and converter B adopts NIDOB strategy.

### Case1: effectiveness of the NIDF strategy

**Figure 12:**
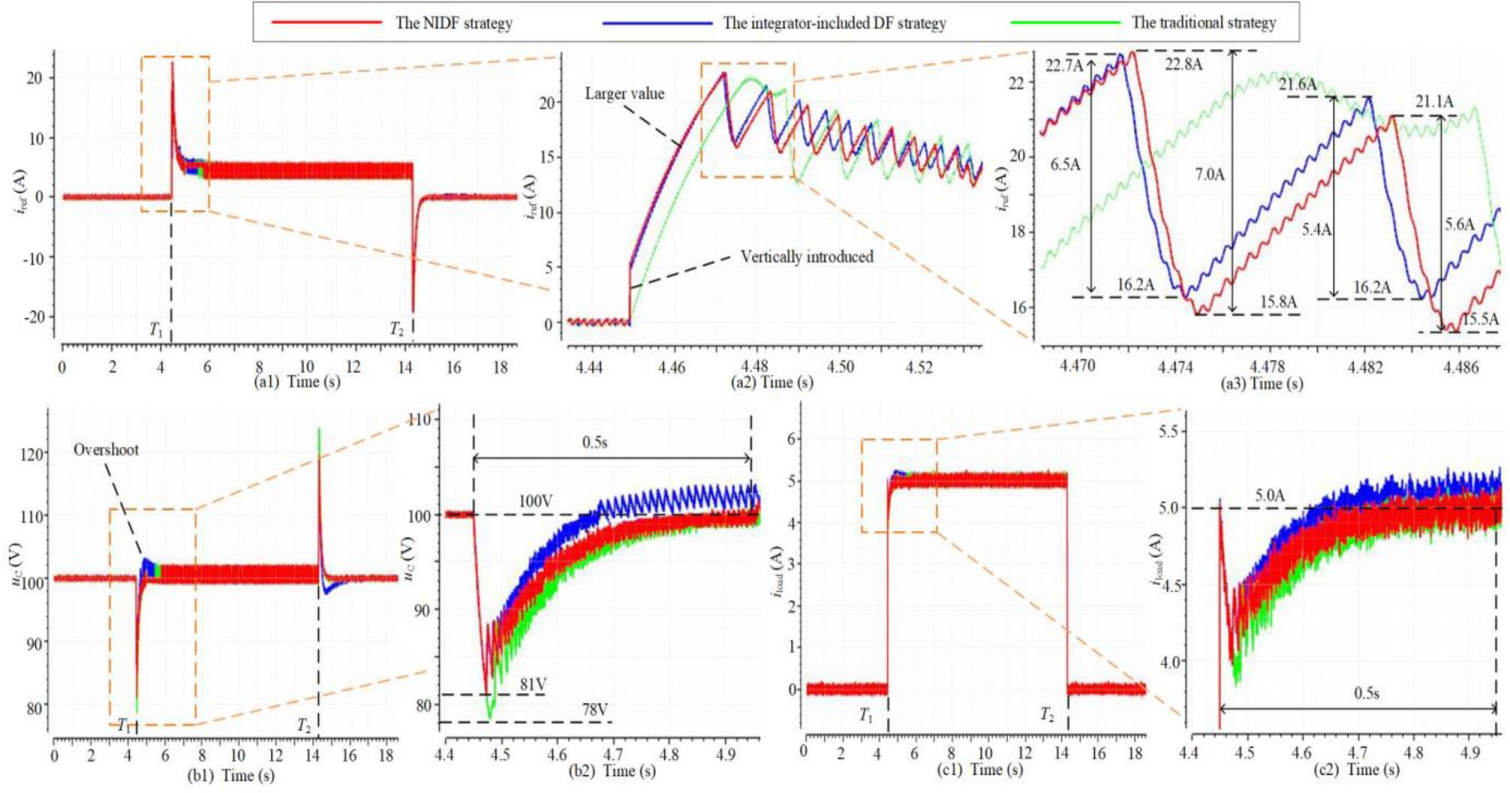
The comparison of the NIDF strategy, the integrator-included DF strategy, and the traditional strategy. (**a**) The waveforms of current reference. (**b**) The waveforms of dc bus voltage. (**c**) The waveforms of load current.

The comparison among the NIDF strategy, the integrator-included DF strategy, and the traditional strategy is shown in Fig. 12, where three GFCs adopt different strategies and 500W loads are separately connected and disconnected to three GFCs at *t* = *T*_1_ and *t* = *T*_2_. From Fig. 12(a1), (b1), and (c1), it can be seen that the three strategies have the same steady current reference equal to the load current, hence all of them can realize CVS. However, in the transient process, the NIDF strategy and the integrator-included DF strategy have a bit faster transient response because they have larger current references than the traditional strategy, as shown in Fig. 12(a2). Besides, since we directly introduce the load current into the current reference, they have an almost vertical increment at the moment of loads being connected. In addition, as shown in Fig. 12(a3), since the integrator can sense the voltage deviation and output a part of the current reference, the integrator-included DF strategy can respond faster than the NIDF strategy at the beginning of the transient process. However, because the output of the integrator has to change continuously, its current reference decreases more slowly than the NIDF strategy as the voltage deviation decreases. Therefore, the integrator-included DF strategy will cause a voltage overshoot, whereas the NIDF strategy can reach the steady-state more quickly. As shown in Fig. 12(b2) and (c2), the NIDF strategy reaches the steady-state after 0.5 s, whereas the overshoot of the integrator-included DF strategy has not fully disappeared yet. Therefore, the NIDF strategy has a better dynamic performance, but it needs an extra sensor to measure the load current.

### Case2: effectiveness of the NIDOB strategy

**Figure 13:**
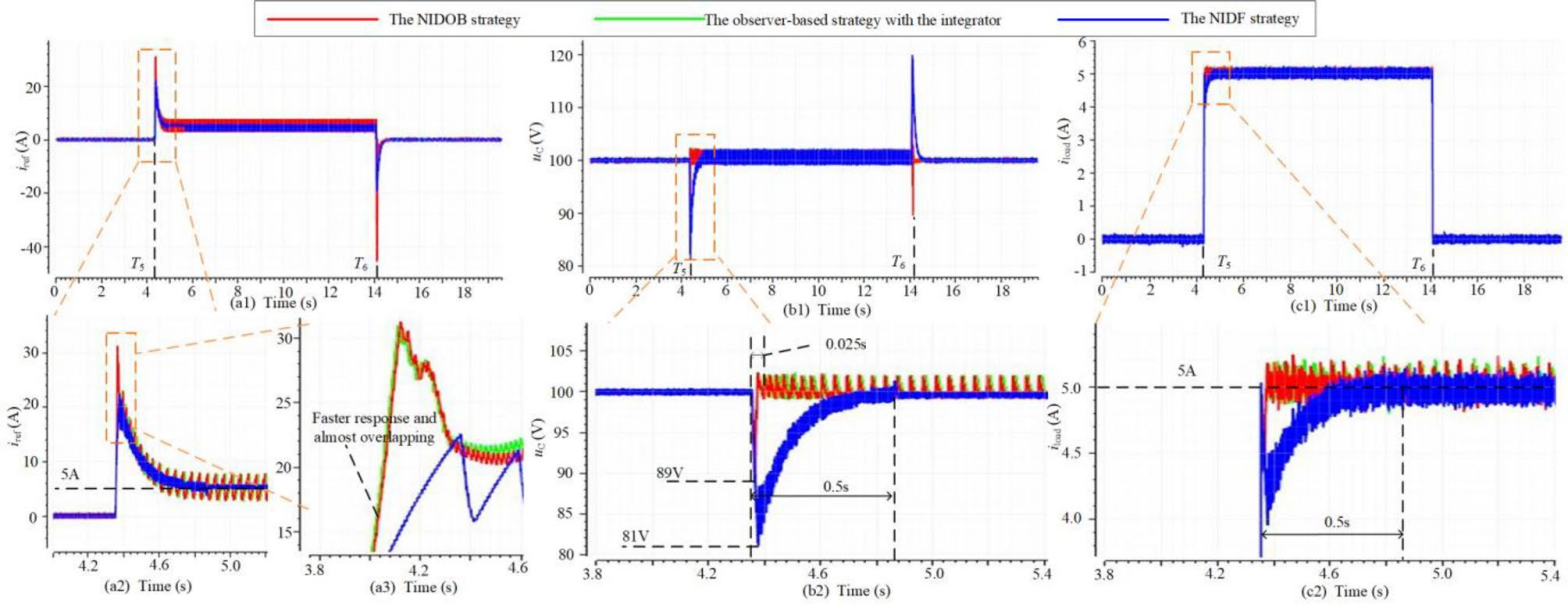
The comparison of the NIDOB strategy, the observer-based strategy with the integrator, and the NIDF strategy. (**a**) The waveforms of current reference. (**b**) The waveforms of dc bus voltage. (**c**) The waveforms of load current.

In Sect. 5 A, the NIDOB strategy has a better dynamic performance. The observer-based strategy with the integrator and the NIDF strategy are compared with it in this case to illustrate the merits of the NIDOB strategy. The loads are connected and disconnected at *t* = *T*_5_ and *t* = *T*_6_, and it can be seen that the NIDOB strategy and the observer-based strategy can generate larger transient current references as shown in Fig. 13(a1)–(a3), hence they have less recovery time and voltage sag as shown in Fig. 13(b1), (b2) and (c1), (c2). As shown in Fig. 13(a2) and (c2), the steady current reference is equal to the load current, hence it is reasonable to directly remove the integrator, which will not influence the realization of CVS. However, since the observation in the observer-based strategy can provide a large enough current reference to make the bus voltage recover very fast, the integrator functions a little and just generates the minor components of the current reference. Therefore, the transient current references of the NIDOB strategy and the observer-based strategy with the integrator overlap with each other (see Fig. 13(a3)), and they have similar dynamic performance (see Fig. 13(b2) and (c2)). Although there is no obvious improvement after removing the integrator, it can simplify the control structure.

### Case3: comparison of the NIDOB strategy and the bandwidth-increased strategy

**Figure 14:**
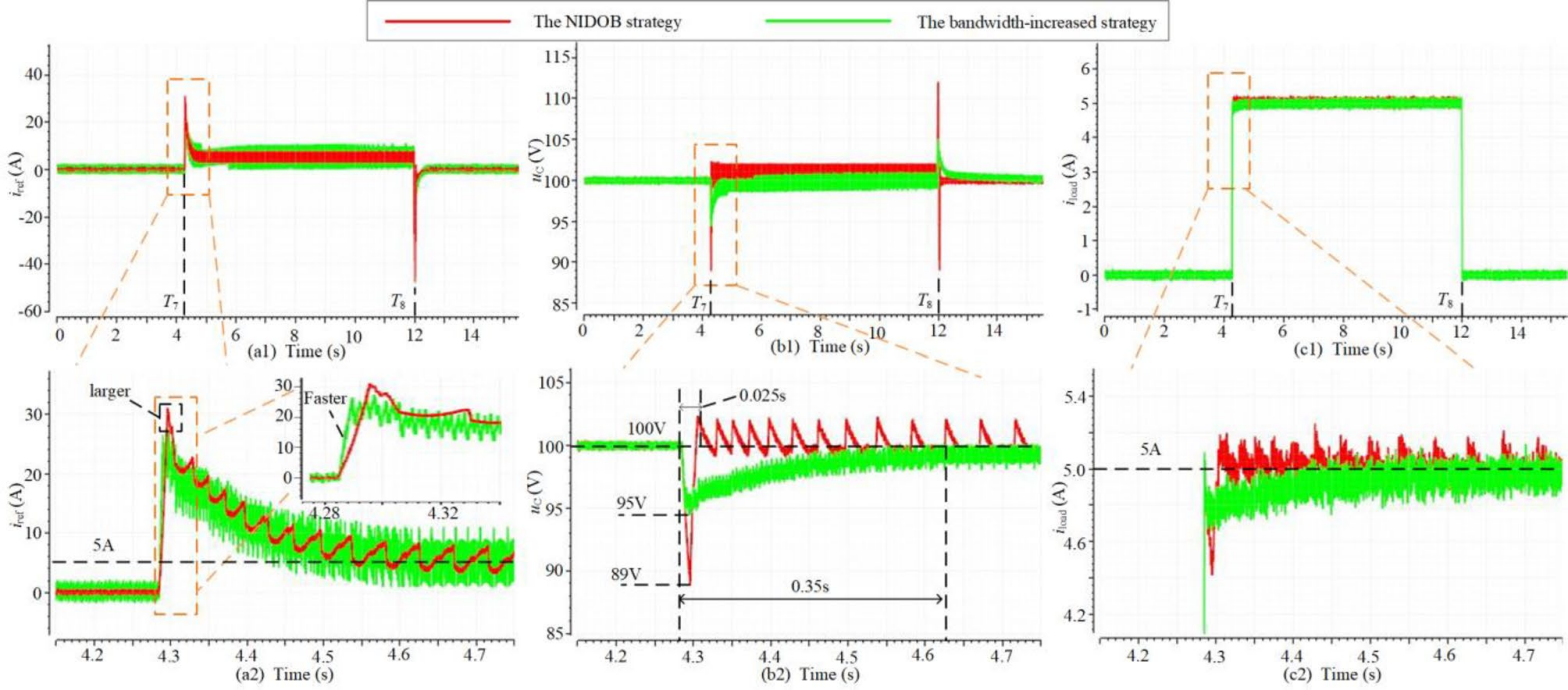
The comparison of the NIDOB strategy and the bandwidth-increased strategy. (**a**) The waveforms of current reference. (**b**) The waveforms of dc bus voltage. (**c**) The waveforms of load current.

To illustrate the advantage of the NIDOB strategy, the bandwidth-increased strategy is compared to it in Fig. 13, where *k*_pu_ = 1 in the NIDOB strategy and *k*_pu_ = 5 in the bandwidth-increased strategy, and the loads are separately connected and disconnected at *t* = *T*_7_ and *t* = *T*_8_. In Fig. 9, the bandwidth of the bandwidth-increased strategy is larger than that of the NIDOB strategy, but its gain over the IF band is smaller than that of the latter. Therefore, the current reference of the bandwidth-increased strategy has a faster response but has a smaller value than that of the NIDOB strategy, as shown in Fig. 14(a1), (a2). Correspondingly, the bandwidth-increased strategy has a smaller voltage sag, but the NIDOB strategy has much less voltage recovery time, as shown in Fig. 14(b1), (b2) and (c1), (c2).

### Case4: Dynamic performance of the NIDOB Strategy under supply voltage disturbance

**Figure 15:**
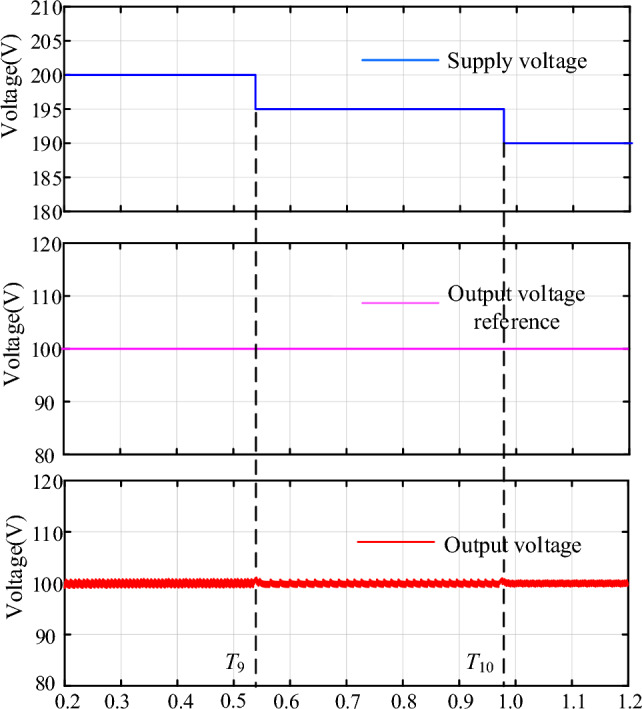
Dynamic performance of the NIDOB strategy under supply voltage disturbance.

To evaluate the dynamic performance of the NIDOB strategy under supply voltage disturbance, the validation is conducted in Fig. 15. It can be seen that the supply voltage decreases to 195 V and 190 V separately at *t* = *T*_9_ and *t* = *T*_10_, but the output voltage waveform only produced small fluctuations. Therefore, the proposed strategy has good performance under supply voltage disturbance.

### Case5: Dynamic performance of the NIDOB Strategy under desired voltage disturbance

**Figure 16:**
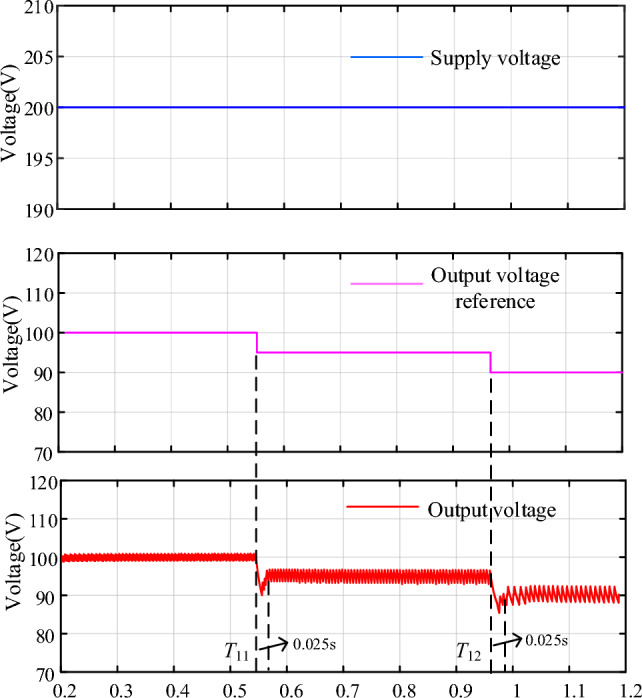
Dynamic performance of the NIDOB strategy under desired voltage disturbance.

To evaluate the dynamic performance of the NIDOB strategy under desired voltage disturbance, the validation is conducted in Fig. 16. It can be seen that the desired voltage decreases to 95 V and 90 V separately at *t* = *T*_11_ and *t* = *T*_12_, but the output voltage waveform only produced small fluctuations. Therefore, the proposed strategy has good performance under supply voltage disturbance.

### Summary

The comparison results of the proposed NIDOB strategy and other strategies are summarized in the following Table 2. It can be seen that the proposed NIDOB strategy has better dynamic performance.

**Table 2:** Comparison results of strategies.

The strategies	The results of compared to the NIDOB strategy
Traditional CV strategy	The NIDOB strategy has faster dynamic performance
Integrator-included DF strategy	The NIDOB strategy has faster dynamic performance
NIDF strategy	The NIDOB strategy has faster dynamic performance
DOB-based strategy with integrator	The two strategies have almost the same dynamic performance, however, the NIDOB strategy has simpler control structure
Bandwidth-increased strategy	The bandwidth-increased strategy has less voltage sag, however, the NIDOB strategy has much less voltage recovery time

## Conclusion

This paper brings forward a NIDOB strategy to improve the dynamic performance of a GFC. This method feeds the load current into the controller to enhance the current reference to accelerate the transient response. In addition, since the feedforwarded value in the steady-state can completely replace the output of the outer integrator, the integrator can be directly removed and the voltage loop can be simply designed as a proportional controller. The proposed method improves the dynamic performance by increasing the gain over the IF band rather than largely increasing the system bandwidth. Compared to the traditional strategy and the NIDF strategy, the NIDOB strategy has better dynamic performance (the recovery time is reduced from 0.5 s to 0.025 s), and it has a simpler control structure than the observer-based strategy with the integrator. Besides, compared to the band-increased strategy, the NIDOB strategy reduces much recovery time without introducing more noises (the recovery time is reduced from 0.35 s to 0.025 s). The theoretical analysis and experimental results can prove its advantages.

The NIDOB method is analyzed and proposed for constant voltage control of DC/DC converter, but it is also promising to employ it inverter’s control, because the AC component can be transformed into DC component through Park's Transformation.

## Data Availability

The datasets used and/or analyzed during the current study available from the corresponding author on reasonable request.
